# Working memory management and predicted utility

**DOI:** 10.3389/fnbeh.2013.00083

**Published:** 2013-07-17

**Authors:** Christopher H. Chatham, David Badre

**Affiliations:** Cognitive, Linguistic & Psychological Sciences, Brown University, ProvidenceRI, USA

**Keywords:** working memory, predicted utility, Q-learning, gating, filtering

## Abstract

Given the limited capacity of working memory (WM), its resources should be allocated strategically. One strategy is filtering, whereby access to WM is granted preferentially to items with the greatest utility. However, reallocation of WM resources might be required if the utility of maintained information subsequently declines. Here, we present behavioral, computational, and neuroimaging evidence that human participants track changes in the predicted utility of information in WM. First, participants demonstrated behavioral costs when the utility of items already maintained in WM declined and resources should be reallocated. An adapted Q-learning model indicated that these costs scaled with the historical utility of individual items. Finally, model-based neuroimaging demonstrated that frontal cortex tracked the utility of items to be maintained in WM, whereas ventral striatum tracked changes in the utility of items maintained in WM to the degree that these items are no longer useful. Our findings suggest that frontostriatal mechanisms track the utility of information in WM, and that these dynamics may predict delays in the removal of information from WM.

## Introduction

Working memory (WM) capacity is limited to as few as 3 or 4 items (Cowan, 2001). Nevertheless, this shallow resource is sufficient to support a wide array of domain-general intellectual abilities (Conway et al., 2003; Ackerman et al., 2005; Cowan et al., 2005; Oberauer, 2005; Kane et al., 2007; Fukuda et al., 2010). Thus, higher cognition requires control systems that can strategically manage our limited WM capacity.

Prior work has identified two key operations that may help to support strategic allocation of WM resources. First, selective updating (i.e., “filtering”) of WM permits only task-relevant information to consume resources (Peers et al., 2005; Vogel et al., 2005; McNab and Klingberg, 2008; Jost et al., 2011). Selective WM updating is thought to rely on striatally-mediated reinforcement learning (RL) systems to identify reward-predictive information in the environment, which then allow only that information into frontally-mediated WM (Braver and Cohen, 2000; Frank et al., 2001; Gruber et al., 2006; McNab and Klingberg, 2008).

Second, a mechanism is required to reallocate WM resources as circumstances change. Consider that information initially judged relevant, and so updated into WM, might later become obsolete or be revealed as entirely irrelevant. Under such conditions, rather than permitting this item to further occupy WM resources, it is adaptive to reallocate WM resources. Much evidence suggests that people can reallocate WM, albeit often sluggishly (on the order of 1–3 s; Oberauer, 2001, 2003, 2005; see also Hasher and Zacks, 1988; Cooper et al., 1996; Hasher et al., 1999; Conway et al., 2003; Cansino et al., 2013; LaRocque et al., 2013). However, relatively little is known about the computational or neurobiological underpinnings of this putative reallocation process.

Both WM updating and WM reallocation could rely on a system for tracking utility. As already noted, WM updating may be supported by RL mechanisms that track the predicted utility of newly-observed information in the world, given some maintained context. Similarly, WM reallocation processes might be supported by RL mechanisms that track the predicted utility of information in WM, given some newly-observed context. Put another way, just as WM updating requires monitoring changes in the utility of information encountered in the environment, WM reallocation could rely on striatally-mediated systems that monitor changes in the utility of information currently maintained in WM. However, there is as yet no evidence that RL plays such a role during WM reallocation.

**Figure 1:**
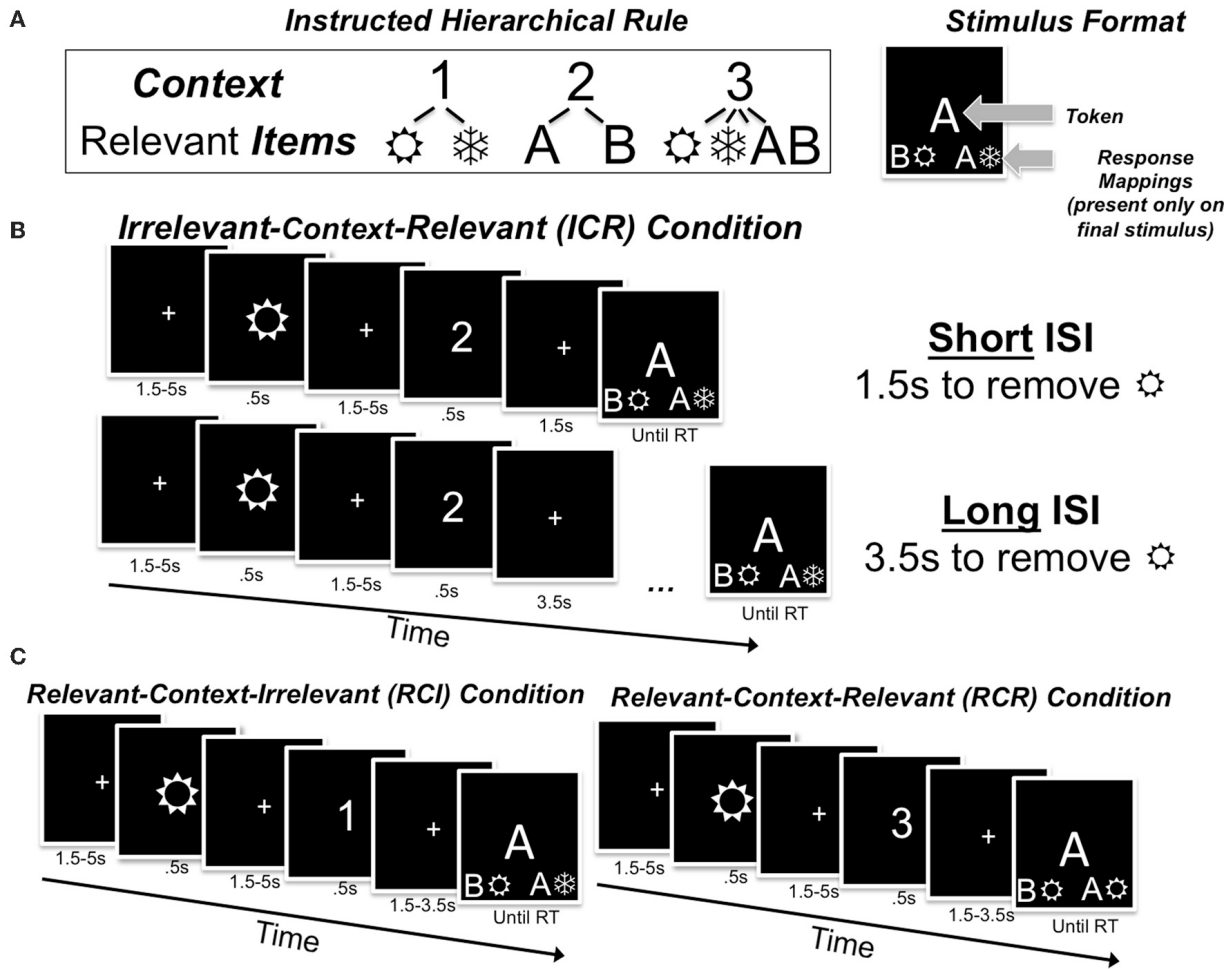
**Task rules (A), theinterstimulus interval manipulation (B), and control conditions (C). (A)** In this sequential WM task, participants are instructed that digits serve as context in determining which class of lower-level items will be relevant for selecting a response. For example, if the digit “1”/“2” appears, wingdings/letters are relevant for responses (respectively), whereas if the digit “3” appears both of the lower-level items will be relevant. Participants are to identify the relevant centrally-presented item(s) from each trial at the bottom of the final stimulus display, and press a corresponding key. **(B)** In the ICR condition, an irrelevant item precedes the context. With a “short” ISI, this irrelevant item may increase the effective WM load; following a longer ISI, the influence of this irrelevant item may be reduced. **(C)** In the RCI control condition, the relevant lower-level item is presented prior to the digit, so participants must merely identify the location of that relevant stimulus at the bottom of the final stimulus display; by contrast, in the RCR control condition, participants may experience a higher effective WM load because both items are relevant for a response.

Here we test the potential role of frontostriatal RL mechanisms during WM reallocation using a novel task. In this task, a disambiguating context was occasionally presented following the storage of a single item in WM. This context, by virtue of an instructed hierarchical rule (Figure 1A), either decreased (Figure 1B), increased (Figure 1C, left), or left mostly unchanged (Figure 1C, right) the predicted utility of the previously-maintained item. After the context was presented, responses were provided to a subsequent item following a variable interstimulus interval (ISI), thereby allowing us to investigate the time course of any behavioral benefits arising from the putative reallocation of WM. Due to the randomized nature of the design, each trial varied in terms of the utility of information in WM following the presentation of the context. Together, these features allowed us to (a) separately assess the behavioral effects of increases and decreases in the utility of information in WM, (b) capture learning on the basis of these changes in an RL model, and (c) correlate these model-based estimates with the BOLD response while participants performed an fMRI-adapted version of the same task.

## Materials and methods

### Participants

Twenty-two right handed adults (aged 18–35; 8 female) with normal or corrected-to-normal vision completed the experiment. All participants were native English speakers and had been screened for the use of psychiatric and neurological medications and conditions, as well as for contraindications for MRI. All participants provided informed consent in accordance with the Research Protections Office at Brown University.

### Basic task design (behavioral)

Each trial consisted of the sequential presentation of a digit (either 1, 2, or 3), a letter (either A or B), and a symbol (the wingdings 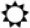 and 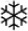). Participants used these tokens to select a left or right key press response based on a hierarchical rule that they were provided at the outset of the experiment (Figure 1A). Specifically, participants were instructed that the digit “1” specified that only the wingding appearing on that trial would be useful for selecting the appropriate response; that the digit “2” specified that only the letter appearing on that trial would be useful for selecting the appropriate response; and that the digit “3” specified that both the letter and wingding appearing on that trial would be useful for selecting a response. Participants selected a response by identifying whether the relevant item (i.e., letter, wingding, or both) appeared on the bottom left or bottom right of the final stimulus event, and pressed the corresponding left or right key. There was always one, and only one, correct answer. In other words, one and only one side of the final stimulus event always contained both lower-level items from any trial involving the digit “3”, or the relevant lower-level item from any trial involving the other digits. Participants were instructed to respond as quickly and accurately as possible upon presentation of the mappings.

Across 180 trials in the experiment, the tokens of each type (letter, symbol, or number) were equiprobable, but appeared in random orders for 500 ms each and with a variable ISI for all stimulus events (1.5–5 s). (Note: The distribution of ISIs was positively skewed to strike a balance between a short task completion time, and our desire to emulate the ISIs that would subsequently be experienced in the scanner.) The randomized presentation complicates the task because digits acting as a higher-level context sometimes occur after lower-level items have already been updated into WM, taxing reallocation mechanisms (Figure 1A).

The key task conditions for the present report are as follows. First, the token presented on the first stimulus event could be rendered irrelevant by the presentation of the subsequent context (i.e., an “irrelevant-context-relevant” trial; ICR; Figure 1B). For example, if a letter was presented first and was then followed by the number “1”, then the letter is irrelevant and the next item (the wingding) will be relevant. Alternatively, the token presented on the first stimulus event could be rendered relevant, either because it was of the class of tokens that were selectively relevant under that context (i.e., a “relevant-context-irrelevant” or RCI trial), or because it was just one of the two tokens that would be relevant on that trial (i.e., a “relevant-context-relevant” or RCR trial; Figure 1C). We note that context could appear in any of the three order positions throughout the task, but we focus on the trials in which it appears in the middle position here.

### Task design (fMRI)

Immediately following completion of the behavioral task, participants were administered the same task in the scanner. The scanner version of the task differed from the behavioral version in three ways: first, participants were instructed to respond with the index and middle fingers of their right hand, as opposed to the index fingers of their left and right hands, corresponding to the left and right sides of the response mappings. Second, the response cues were provided as a separate stimulus event that followed the last token on each trial by the same variable interval used between all other stimuli in the scanned version of the experiment (1.5–9.5 s). This delay between the final stimulus event and the response mappings event would be expected to mask the behavioral effects indicative of reallocation because they could occur during the interval itself. Consequently, it was not possible to assess the same behavioral effects in the scanner, although performance in the scanner remained comparable to that observed in the behavioral session (see Results, below) Finally, the task was administered in 4 × 10-min blocks, rather than one continuous block.

Unlike the behavioral version of the experiment, the duration of each interstimulus and intertrial interval was determined by a variant of OptSeq2 (Greve, 2002), under the constraint that no more than 33% of the total time spent in the scanner consisted of null fixation events, so as to optimize the design for use with rapid-event related fMRI. Stimulus duration was kept at a constant 500 ms. The optimized fMRI design yielded 24, 18, and 25 individual trials for the ICR, RCI, and RCR conditions for each subject, respectively.

### Reinforcement learning model

The statistics of this task are such that the presentation of context in the ICR and RCI conditions would be expected to decrease and increase (respectively) the predicted utility of the preceding item in WM. By contrast, the RCR condition yields less change in the predicted utility of the preceding item. This approximate symmetry occurs because, while there is always one and only one correct response for each trial, 50% of the time the *incorrect* response option also involved one item that had been seen on that trial (a “lure” trial). We formalize these notions in our RL model such that for each item, *i*, occurring on a trial, *t*, the experienced utility *U* is determined by the item's association with the correct response:

(1) $$U_{t,i}=\{\begin{array}{ll} 1 & \text{if associated with correct response}\\ 0 & \text{otherwise} \end{array}$$

Concretely, an item is considered to be “associated” with the correct response if that item appeared only on the side of the screen corresponding to participants' button press, and that button press was objectively correct.

For each correct trial the context, *c*, is also given a utility:

(2) $$U_{t,c}=\{\begin{array}{ll} 1 & \text{if context is necessary to determine correct response}\\ 0.5 & \text{contextis unnecessary to determine correct response} \end{array}$$

Context was considered “unnecessary” to determine the correct response on non-lure trials, because the same correct answer would have been selected regardless of which context had appeared on that trial.

The predicted utility, *PU*, of each item was updated across trials according to a utility prediction error, scaled by the learning rate, α:

(3) $$PU_{t+1,\;i}=PU_{t,i}+\alpha \underset{“\text{UPE}”}{\underset{︸}{\left(U_{t,\;i}-PU_{t,i}\right)}}$$

The identical equation was applied for determining the predicted utility of each context, replacing “*i*” with “*c*” in the equation above.

For simplicity, UPE was set to zero for any item or context that would not presently be in WM at the time of a response, assuming participants followed the instructed rules and were capable of removing that information from WM; it was also set to zero for any items and contexts appearing on incorrect trials.

To fit the observed RT for the ICR short ISI condition (see Equation 4), the most likely learning rate and starting utilities for items and contexts was found for each participant via a hierarchical Bayesian model implemented in JAGS (Just Another Gibbs Sampler, Plummer, 2004) for R (CRAN Project for Statistical Computing). In this model, each fitted parameter for each participant was simulated as a random draw from a normal distribution characterizing that parameter across population; these hyperparameters were given flat hyperpriors. 3 chains of Gibbs samples were burnt-in for 1000 samples, with an additional 10,000 iterations of Gibbs samples used for *a posteriori* inference. The posterior means were used as point estimates for each participant.

### MRI procedure

Whole-brain imaging was performed on a Siemens 3 T TIM Trio MRI system with a 32-channel head coil. A high resolution T1 multi-echo MPRAGE was collected from each participant. Functional images were acquired in four runs, each consisting of 303 volumes, with a fat-saturated gradient-echo echo-planar sequence (*TR* = 2 s, *TE* = 28 ms, flip angle = 90°, 38 interleaved axial slices, 192 mm FOV with voxel size of 3 mm^3^). Head motion was restricted with padding. Visual stimuli were rear-projected and viewed with a mirror attached to the head coil. Participants responded using an MRI-compatible button box.

### Data preprocessing

#### Behavioral data

The first 10 trials of the behavioral experiment were excluded, as were any incorrect trials or trials with RTs more than 5 standard deviations from a participant's mean RT.

#### Imaging data

Data were processed using a combination of SPM and FSL. First, SPM8 tools (artglobal and tsdiffana) were used for artifact detection, and slice timing correction was then performed. The first six volumes of each run were discarded to allow the scanner to reach steady state. The data were motion-corrected using rigid transformations in MCFLIRT to the middle acquisition of each run. Only one participant moved more than 2 mm within any run, so that one run was excluded from analysis. Grand-mean intensity normalization of the entire 4 D dataset was performed with a single multiplicative factor, and the data were subjected to a temporal highpass filter (Gaussian-weighted least-squares straight line fitting, with sigma = 32.5 s), and the data were smoothed at 8 mm FWHM. The middle acquisition of each run was then registered to each participant's brain-extracted MPRAGE using a linear 7DOF transform, and the MPRAGE was registered to the MNI standard brain using a linear 12DOF transform.

### Statistical analysis

For behavioral data, accuracy and reaction time on correct trials for the three conditions (ICR, RCI, and ICR) at each ISI [short (1.5 s) vs. long (3.5 s)] for each subject were averaged within each cell of this 3 × 2 design, and subjected to two-tailed tests respecting non-independence in the data (e.g., paired-sample *t*-tests, repeated measures ANOVAs, or the equivalent appropriate test). Too few trials were included at the longest ISI of 5 s to permit analysis of that cell alone, so those trials were excluded. Where ANOVA is used, we report single degree-of-freedom *F*-tests for the focused contrasts of interest as estimated through orthogonal contrast codes (e.g., Helmert coding; Rosenthal and Rosnow, 1985). Error rates were subject to the variance-stabilizing arcsin square root transformation prior to analysis. All error bars represent ±1 standard error.

For the fMRI analysis, a GLM was estimated using FEAT (FMRI Expert Analysis Tool) version 5.98 (FMRIB's Software Library, www.fmrib.ox.ac.uk/fsl), on the basis of explanatory variables (EVs) coding for the following event types: the selective contextual cue (i.e., Digits 1 or 2) or the global contextual cue (i.e., Digit 3) appearing in the first position, middle position, or last position; and items appearing in the first or middle position. Separate EVs were used to capture items appearing in the last position under the selective context and global contexts for reasons that are unimportant for the present report. The duration of each event in all these EVs was set to the stimulus duration (500 ms). Separate boxcar EVs were used to capture responses; the duration of each event in these EVs was set to the observed RT on each trial. Additional nuisance EVs were also included in the GLM, including those corresponding to trials where participants responded incorrectly (although accuracy was uniformly above 90% for each condition as performed within the scanner), and to the 6 degrees of movement estimated by MCFLIRT. All EVs except those corresponding to movement were convolved with a standard hemodynamic response function, high-pass filtered in the same way as the functional data, and then used as a regressor (including temporal derivatives) in the GLM.

Where small-volume correction for the basal ganglia is used, the mask used consisted of the anatomically defined caudate, nucleus accumbens, putamen, and pallidum, as defined according to the Harvard-Oxford subcortical atlas.

### Model-based fMRI analyses

The learning rates and predicted utilities for each item and context at the conclusion of the behavioral experiment were used as the learning rates and predicted utility values for each item and context for an identical RL model applied to the trials experienced by each participant in the scanner. In other words, the RL model was fit to data from the behavioral experiment, and the fitted model was then applied to the sequence of trials experienced by that same subject during the fMRI experiment. The resulting trial-by-trial predicted utility estimates were used to calculate the same composite term that correlated with behavior outside the scanner. Specifically, the composite term was computed using the expression for predicted utility included in Equation 4, below. This term was then used as a trial-by-trial parametric regressor, normalized to a mean of zero and standard deviation of one within each run, for the ICR condition. Equivalent terms for the RCI and RCR conditions were also calculated, and used as trial-by-trial parametric regressors for comparison purposes. Each instance of those conditions was modeled as an event onsetting with the context, lasting 0.5 s in duration, after convolution with a standard hemodynamic response function (and its temporal derivative).

## Results

### Overall performance during “context middle” conditions

The present report focuses on the trials during which the disambiguating context appeared in the middle position, though participants encountered other orderings during the task, as well (see Materials and Methods). Performance on these “context middle” events was high overall. Specifically, the mean error rate was 8.9%, with a mean RT of 819 ms during the behavioral portion of the experiment. Although performance remained high during the scanned version of the task (mean error rate of 8.1%, mean RT of 803 ms), our further behavioral analyses focus entirely on the behavioral portion of the experiment, where the phenomena indicative of WM reallocation would not be masked by the longer ISIs used in the scanned version of the experiment (see Materials and Methods).

### Effects of ISI on performance (behavioral experiment)

If participants do reallocate WM resources when the predicted utility of a maintained item suddenly drops (as in the ICR condition; Figure 1B), and such reallocation is time consuming, then performance should be affected by the amount of time available for reallocation. Accordingly, we divided the ICR, RCI, and RCR trials depending on whether a short ISI (1.5 s) or a long ISI (3.5 s) was provided between the presentation of the context and the final stimulus event + response mapping. In this way, we assessed changes in performance as a function of the time elapsed after the presentation of the disambiguating context.

Performance differed between the RCI and the RCR condition comparably across both long and short ISIs. Specifically, the RCI condition was associated with reduced RT [*F*_(1, 21)_ = 84.8, *p* < 0.001], but somewhat more errors [*F*_(1, 21)_ = 4.7, *p* = 0.04], than the RCR condition, independent of ISI. The difference in RT across conditions, irrespective of ISI, was not explained by the difference in accuracy between the conditions (as indicated by a significant effect of condition [RCR vs. RCI] even after controlling for the difference in accuracy between the conditions; *F*_(1, 20)_ = 67.98, *p* < 0.001), nor was there any correlation between the difference in RT and the difference in accuracy (Pearson R = 0.0006), both results arguing against a simple speed-accuracy tradeoff. As the maintained first position item is useful in both of these conditions, this disproportionate increase in RT may in part be attributable to the added WM load from the third position item in the RCR condition (though other factors may also contribute to the elevated RT in this condition).

**Figure 2:**
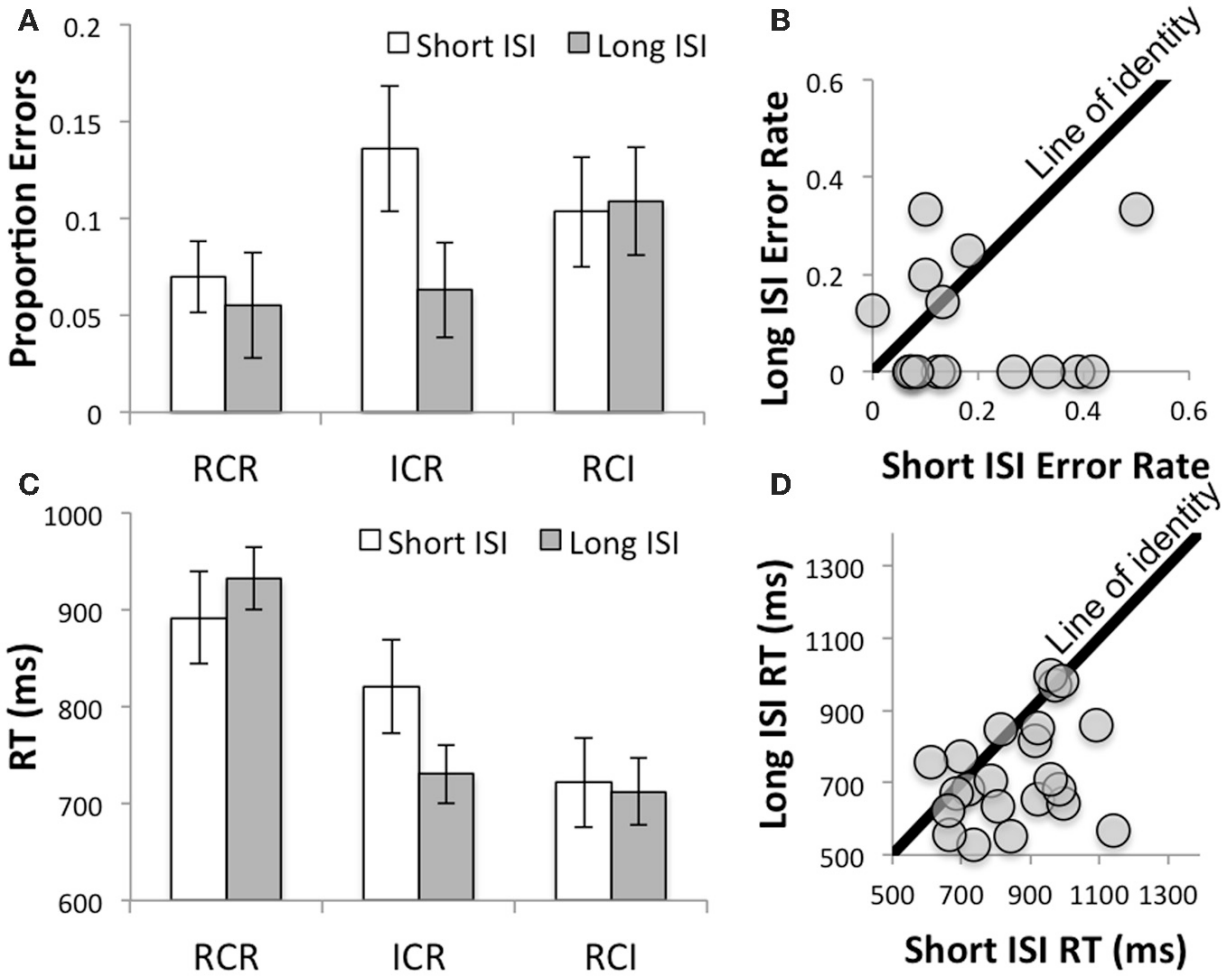
**Disproportionate benefits as a function of ISI in the ICR condition in terms of errors (A and B) and reaction times (C and D). (A)** Error rates decreased as a function of ISI in the ICR condition. **(B)** The majority of the data fell reliably below the line of identity relating error rates on the short and long ISI trials in the ICR condition, indicating a reliable increase in accuracy as a function of ISI in that condition. [Note: participants with perfect accuracy across both ISIs are not shown (*n* = 6)]. **(C)** As with Errors, RT decreased as a function of ISI in the ICR condition, relative to the control conditions. **(D)** Again, the majority of the data fell below the line of identity relating RT on the short and long ISI trials in the ICR condition, indicating a reliable decrease in RT as a function of ISI.

Nonetheless, and consistent with the idea that participants undertake a time-consuming reallocation process, performance was differentially improved at the longer ISI in the ICR condition relative to the other conditions (in terms of the interaction of ISI with the focused contrast of ICR vs. the other conditions: *F*_(1, 21)_ = 3.17, *p* < 0.09 and *F*_(1, 21)_ = 9.0, *p* = 0.007 for errors and RT, respectively). This interaction was further probed with focused *t*-tests, indicating that the longer ISI was associated with improved performance in the ICR condition [*t*_(21)_ = 2.62, *p* < 0.02 and *t*_(21)_ = 3.56, *p* = 0.002 for errors and RT, respectively), but not in the RCI or RCR conditions (*t*'s < 1.2, *p*'s > 0.25 and *t*'s < 1.4, *p*'s > 0.15 for errors and RT, respectively). Figure 2 depicts these effects in terms of errors (**A** and **B**) and reaction times (**C** and **D**), both at the level of the group means (**A** and **C**) and for individual subjects (**B** and **D**). Thus, these results suggest that at a short ISI, the lingering effect of the not-yet-removed first position item produced a higher WM load effect, akin to that which might contribute to the poorer performance in the RCR condition^1^. By contrast, with sufficient time, the loss of the first item may reduce effective WM load, yielding performance that is more comparable to the RCI condition.

### Effects of response lures on performance (behavioral experiment)

One potential concern is that the aforementioned effects were due to a differential effect of response lures in the ICR condition. Specifically, it could be that priming or potentiation of a recently maintained item at the short ISI made it more difficult to reject the response that was cued by that irrelevant item. Thus, the performance declines at the short ISI might arise from this response competition effect, rather than a load effect. However, there was no evidence of increased sensitivity to lures that would be predicted by this account. In particular, the critical interaction of ISI with condition remained significant even when controlling for response lures [*F*_(1, 21)_ = 7.84, *p* = 0.01]. The only effects attributable to lures were an increase in RT in the RCR condition [*F*_(1, 21)_ = 12.54, *p* = 0.002], and a slight elongation of RT at short, relative to long ISIs across all conditions [*F*_(1, 21)_ = 3.29, *p* < 0.09], with no other effects approaching significance (all *F*'s < 1.71, *p*'s > 0.2). Thus, the disproportionate benefit of ISI in the ICR condition was not attributable to the effect of lures.

### Reinforcement learning model fit (behavioral experiment)

Having observed a differential benefit of ISI on performance in the ICR condition in terms of RT, we next assessed whether trial-to-trial variations in this condition's RT would correlate with model-based estimates of the predicted utility of the irrelevant first item. To this end, we tested whether the speed with which participants could respond correctly on ICR trials involving a short ISI could be predicted as a linear function of the predicted utility of the irrelevant item, *PU*_*irrel*_, scaled by the inverse utility of the context:

(4) $$RT_{ICR\;Short\;ISI}=B_{0}+B_{1}PU_{irrel}\left(1-PU_{c}\right)+\varepsilon$$

**Figure 3:**
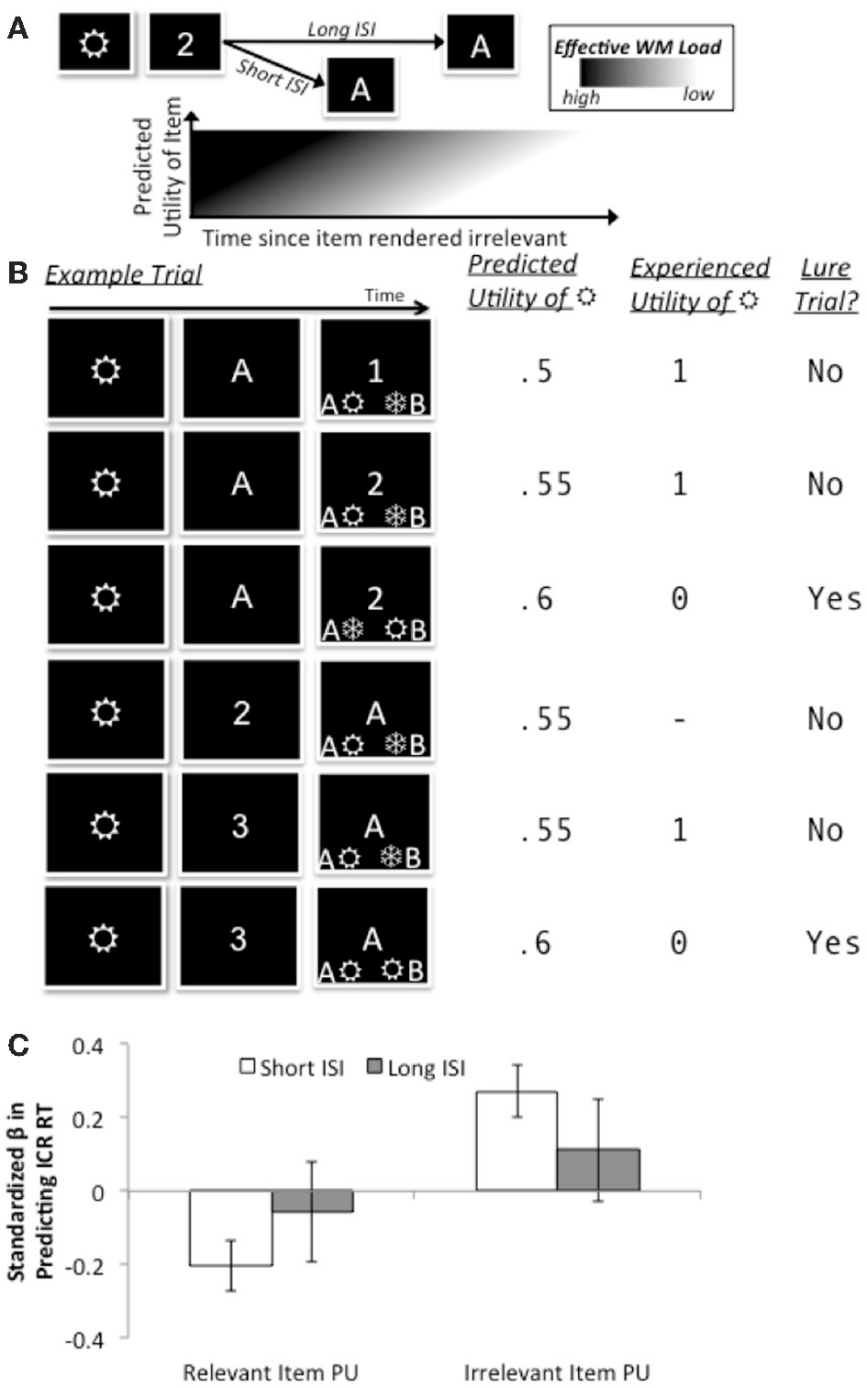
**Conceptual schematic of computational model (A), trial-by-trial RL-like updating of utilities (B), and significant fits to RT in the ICR condition as a function of ISI (C). (A)** The key conceptual assumption in our model is that the benefit of a longer ISI to RT and accuracy in the ICR condition reflects a sluggish reduction in effective WM load following the presentation of context, but that this sluggishness is exacerbated for irrelevant items that had a higher predicted utility. **(B)** Several examples illustrate how predicted and experienced utilities for an example item (“
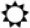
”) change across trials. In the first illustrated trial, 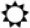 is specified as relevant by the context and is associated with the correct response, therefore acquiring an experienced utility of 1; the predicted utility on the following trial is updated by the resulting utility prediction error, scaled by the learning rate (here, 0.09). On this second trial, “
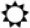
” is specified as irrelevant (due to the context) but is still associated with the correct response, and thus again acquires an experienced utility of 1. This experience changes its predicted utility for the third trial, where it is now associated with the incorrect response, and acquires zero experienced utility. In the fourth trial, 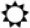 is not eligible for experienced utility because it should not be held within WM (the context had rendered it irrelevant), whereas in the fifth and sixth trials, R is associated/not associated (respectively) with the correct response and acquires utilities of 1/0, respectively. **(C)** At the short ISI of the ICR condition, the predicted utility of irrelevant items was positively correlated with RT (“irrelevant utility cost”), whereas the predicted utility of the relevant items was negatively correlated (“relevant utility benefit”). These effects were diminished at the long ISI.

The basic prediction underlying this model is that performance in the short ISI condition is poor because of a higher effective WM load caused by the maintenance of the irrelevant item. This higher load will decrease more slowly in time for items with higher predicted utility, leading to a differential cost for those items on short ISI trials (see Figure 3A). Figure 3B illustrates how predicted utility and experienced utility of a lower-level item would change as a function of various trial characteristics; as noted in Materials and Methods, lower-level items are granted a experienced utility of 1 only if they would be maintained in WM upon presentation of the final stimulus event, and they are uniquely associated with the correct response.

The resulting trial-by-trial estimates of predicted utility showed a reliably *positive* correlation with RT on the short ISI trials (one sample *t*-test against zero: *t*_(21)_ = 3.84, *p* = 0.001), even though neither the sign nor significance of this relationship was enforced by the model fitting procedure. In other words, RTs on short ISI trials in the ICR condition were significantly longer when the irrelevant item in memory had a higher expectation of being associated with the correct response (i.e., predicted utility). Interestingly, this “irrelevant utility cost” on RT was complemented by the nearly-equal and opposite “relevant utility benefit” to RT, arising from the predicted utility of the final item in ICR trials (one sample *t*-test against zero: *t*_(21)_ = 2.97, *p* = 0.007). In addition, similar but apparently diminished effects were observed at the long ISI (*t*'s < 1.08, *p*'s > 0.29; Figure 3C).

### Task-based fMRI

#### Context vs. fixation

**Figure 4:**
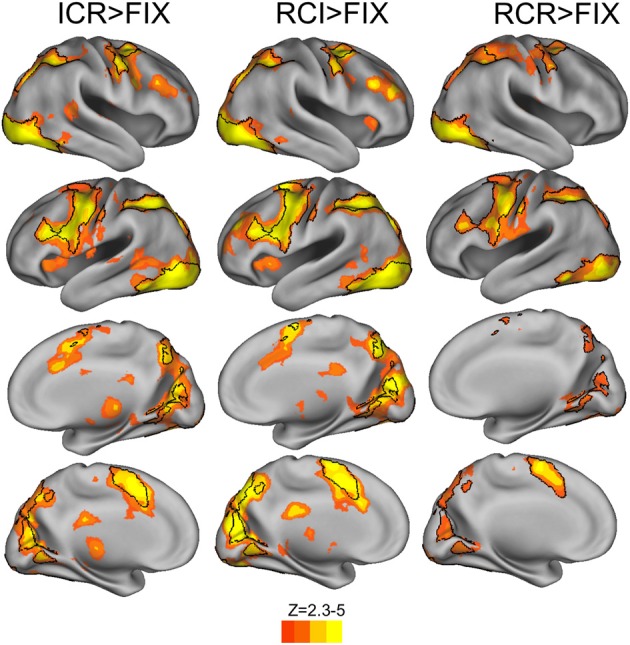
**BOLD responses to context vs. fixation (red-yellow) and common areas of recruitment (black outlines)**. Relative to fixation, each of the three conditions elicited a reliable BOLD response across frontal, parietal, and occipital cortex. Most significant frontal activation, whether medial and lateral, was observed in the left hemisphere (voxelwise *z* > 2.3, corrected to *p* < 0.05 via GRF).

As a first step in assessing whether the unique behavioral effects identified in the ICR condition might also be associated with unique hemodynamic patterns, we first contrasted the BOLD response to context in the ICR, RCI, and RCR conditions with fixation. These contrasts yielded robust activity throughout a frontoparietal network (Figure 4). Consistent with prior work using 2nd order hierarchical rule tasks like this one, several regions in this network were commonly activated across all three conditions (black outlined regions of Figure 4), including intraparietal sulcus, dorsal premotor (PMd) cortex in the right hemisphere and numerous left prefrontal regions, including dorsal PMd, pre-premotor (pre-PMd), and inferior frontal sulcus (IFS).

#### Task-based fMRI: contrasts of responses to context across conditions

**Figure 5:**
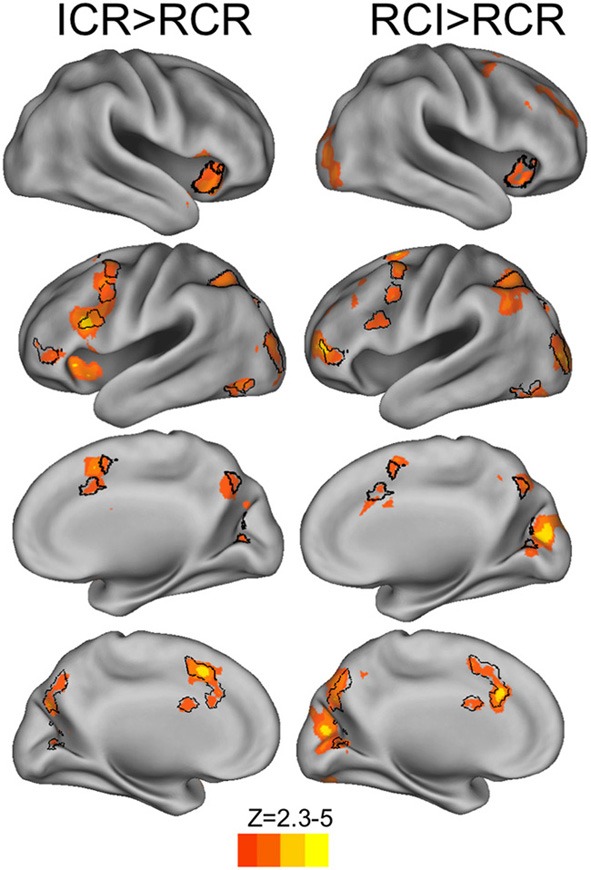
**BOLD responses to selective vs. global context conditions (red-yellow) and common areas of recruitment (black outlines)**. Contrasts of RCI and ICR vs. RCR revealed reliable BOLD response across frontal, parietal, and occipital cortex (voxelwise *z* > 2.3, corrected to *p* < 0.05 via GRF). The reverse contrasts of RCR > ICR and RCR > RCI, and the direct contrasts of ICR with RCI, failed to reach significance.

Direct comparisons of the BOLD response across conditions revealed widespread increases in BOLD at the onset of context in both the ICR and RCI conditions relative to the RCR condition (Figure 5, red-yellow regions), including within the IFS, pre-PMd, PMd, intraparietal sulcus, dorsomedial prefrontal cortex, right anterior insula, and left inferior frontal gyrus. Several of these regions were recruited across both the ICR and RCI conditions more strongly than in the RCR condition (Figure 5, black outlines), including right anterior insula, left IFS, pre-PMd, PMd and bilateral dorsomedial prefrontal cortex. Nonetheless, the only significant difference observed in a direct contrast of the ICR and RCI conditions was a small cluster of voxels in primary visual cortex, which showed only a modest increase in recruitment during the RCI than ICR condition. There were no indications of an increased response during the ICR vs. RCI anywhere in frontoparietal or striatal areas, even at a liberal threshold (*p* < 0.05 uncorrected).

To assess whether any potential BOLD differences between the ICR and RCI conditions might have been obscured by an ill-fitting canonical hemodynamic response function, we utilized FMRIB's Linear Optimized Basis Sets (FLOBS; Woolrich et al., 2004) to reconstruct the hemodynamic timecourse for each condition in each of multiple ROIs taken from Figure 5. However, in no case were significant differences observed between the ICR and RCI conditions—not in terms of peak signal change, integrated signal change, or time to peak. Instead, both the ICR and RCI conditions evinced fairly similar timecourses across a variety of regions.

Thus, we found a commonly observed frontoparietal control network to be engaged during performance of the task. However, this network only responded differentially to selective (RCI/ICR) vs. global (RCR) updating conditions, and did not further differ within the selective conditions. Likewise, there were not other regions of the brain that distinguished between these conditions. Hence, in the present design, we did not find evidence of a unique system supporting active removal from WM. Next we sought to identify the source of the predicted utility effects on WM reallocation.

#### Model-based fMRI: trial-by-trial predicted utility regressor

The above analyses indicate that while the ICR condition is distinguishable from the other conditions behaviorally, the task-induced BOLD response does not clearly differentiate the ICR condition from the others. However, our computational model offers an additional opportunity to distinguish these conditions, because the same model fit to the behavioral experiment can be applied to the sequence of trials experienced by those same subjects in the fMRI experiment (see Materials and Methods). The resulting trial-by-trial estimates of the predicted utility of the items putatively residing in WM were used as parametric regressors at the onset of the context events, to determine whether the ICR condition might be distinguished from the others in terms of the BOLD response to these fluctuations in predicted utility.

**Figure 6:**
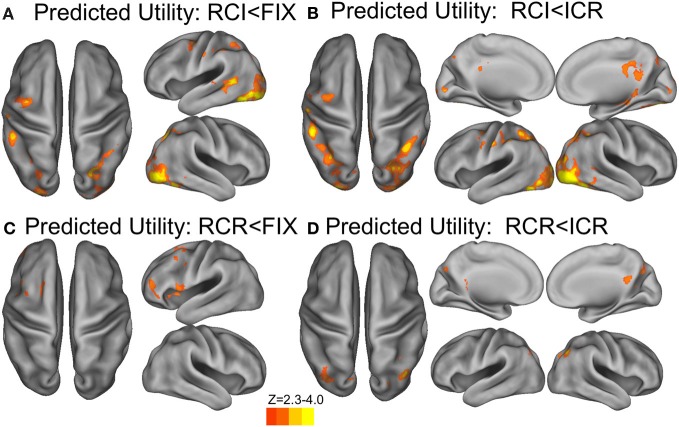
**BOLD correlates of model-based Predicted Utility estimates**. In the RCI condition, trials associated with lower predicted utility at the presentation of context elicited a reliably stronger BOLD response in visual cortex, anterior intraparietal sulcus, and a region of dorsal premotor cortex, both relative to fixation **(A)** and relative to the ICR condition **(B)**. A similar effect was observed in scattered sections of the prefrontal cortex for the RCR condition, including left rostrolateral prefrontal cortex, left dorsal premotor cortex, and ventral prefrontal cortex **(C)**, although only activation in the occipital lobe differentiated these effects of RCR from the ICR condition **(D)**. Voxelwise *z* > 2.3, corrected to *p* < 0.05 via GRF.

**Figure 7:**
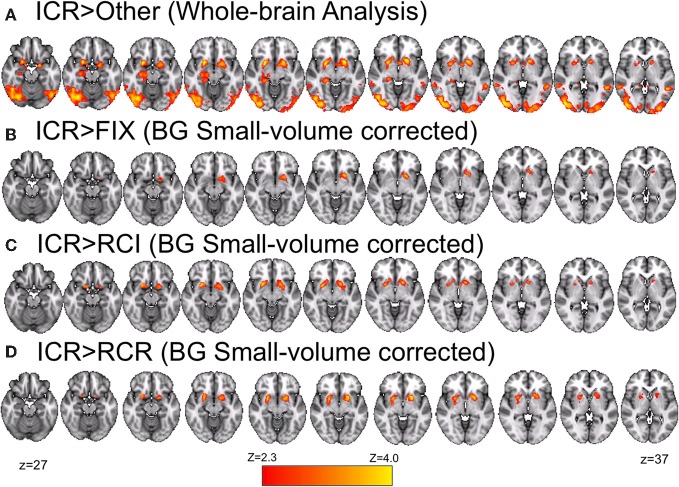
**Ventral striatal BOLD positively and differentially correlates with the predicted utility of items in WM in the ICR condition. (A)** Relative to all other conditions, bilateral ventral striatum was more strongly correlated with the predicted utility of items in WM in the ICR condition. **(B)** After restricting our analyzed volume to the basal ganglia (all other regions masked), left ventral striatum was found to show a positive correlation with the predicted utility of items in WM in the ICR condition. No other condition showed this effect, and indeed bilateral ventral striatum was more positively correlated with predicted utility in the ICR condition than either the RCI **(C)** or RCR **(D)** conditions alone. Note that **(B–D)** mask out activity outside of the basal ganglia (including caudate, nucleus accumbens, pallidum, and putamen). Voxelwise *z* > 2.3, corrected to *p* < 0.05 via GRF.

In a whole-brain analysis, fluctuations in predicted utility reliably predicted BOLD decreases in the RCI condition (Figure 6A), differentially so relative to the ICR condition (Figure 6B), in parts of dorsal PMd cortex, anterior intraparietal sulcus, and visual cortex. Similarly, fluctuations in predicted utility also reliably predicted BOLD decreases in the RCR condition (Figure 6C), again differentially so relative to the ICR condition (Figure 6D), although the latter effect was limited largely to the visual cortex and posterior cingulate. No regions were found to significantly correlate with trial-by-trial fluctuations in the predicted utility of information in WM in the ICR condition, relative to fixation. A whole-brain contrast of the ICR vs. the other conditions yielded differences not only in visual cortex and posterior cingulate, in line with the above contrasts, but also the left hippocampus and bilateral ventral striatum (Figure 7A).

We further interrogated these effects by focusing our analysis on the basal ganglia. Small-volume correction revealed a positive correlation of left ventral striatum with trial-by-trial fluctuations in the predicted utility of the preceding item in the ICR condition alone (Figure 7B). In bilateral ventral striatum, this positive correlation during the ICR condition was reliably stronger than that observed in both the RCI (Figure 7C) and RCR (Figure 7D) conditions. By contrast, no significant correlation was observed in either the RCI or RCR conditions in these small-volume corrected analyses.

## Discussion

Here we provide evidence that the allocation of WM resources is influenced by the predicted utility of to-be-remembered items, and we implicate the ventral striatum in coding this historical utility during the reallocation process. Specifically, WM task performance was disproportionately impaired at short latencies after the presentation of a disambiguating context, if that context specified a preceding item as irrelevant for an upcoming response. This impairment scaled with the historical utility of these putatively-irrelevant items. A greater frontoparietal BOLD response was elicited by larger changes in predicted utility (i.e., the RCI and ICR conditions) than smaller changes (the RCR condition), but neither its amplitude nor shape depended the sign of the change [whether positive (RCI) or negative (ICR)]. Further, in dorsal PMd cortex, anterior intraparietal sulcus, posterior cingulate, and visual cortex, BOLD negatively correlated with trial-by-trial fluctuations in the predicted utility of items that should continue to be maintained. BOLD in the ventral striatum, by contrast, positively correlated with trial-by-trial fluctuations in the predicted utility of items that were previously relevant but should *no longer* be maintained.

These results are consistent with proposals that frontal cortex and striatum dissociably support WM maintenance vs. updating, respectively (e.g., Barch et al., 1997; Fukai, 1999; Frank et al., 2001; Miller and Cohen, 2001). By these accounts, recurrent networks of cells in lateral frontal cortex support robust maintenance of items in WM (e.g., Durstewitz et al., 2000; Sakai et al., 2002). Here, dorsal PMd activity correlated with items' predicted utility only in contexts requiring the continued maintenance of these items (i.e., the RCI and RCR conditions). Thus, not only is lateral frontal cortex engaged by contexts that require continued maintenance, but this maintenance-related activity is modulated by the utility of the items. In contrast, striatum has been hypothesized to support an updating or a “gating” function for updating WM via thalamo-cortical disinhibition (Frank et al., 2001). Consistent with this gating function, striatum correlated with the predicted utility of items only in contexts specifying that WM resources should be allocated away from a previously-maintained item. Thus, whereas utility modulated PFC activation under conditions requiring maintenance, utility modulated striatal activation under conditions requiring gating. However, the “gating” response observed here is not evoking the selection of a new item to put in WM, in any obvious way. Rather, this utility-driven response in striatum is involved in a reallocation process, such as selecting a plan to update an upcoming item, dropping the active maintenance of an item in memory, deprioritizing this item, or perhaps even actively removing the obsolete item. We return to the question of mechanism below.

The modulation of distinct frontal WM maintenance vs. striatal gating functions by predicted utility could parallel the distinct actions of dopamine on frontal vs. basal ganglia systems (Durstewitz et al., 2000; Cools, 2008). Specifically, evidence from animals and humans has suggested that dopamine enhances “stability” in frontal cortex, making WM maintenance more resistant to distraction. By contrast, increased dopamine in striatum enhances “flexibility,” facilitating switching to new responses or courses of action as circumstances demand. Similar to the frontostriatal correlates of predicted utility reported above, distinct BOLD changes have been evoked in PFC vs. maintenance vs. task switching, respectively, by dopamine agonists (e.g., bromocriptine; Cools et al., 2007). If the encoding of expected value by dopaminergic projections to striatum and PFC also encodes the historical value of items in WM (Frank et al., 2001), then dopamine might mediate the frontostriatal correlates of predicted utility observed here. This hypothesis makes clear predictions for the effects of dopaminergic drugs on this task.

Pharmacological tests might also help establish the causality among the frontostriatal mechanisms we implicate in encoding predicted utility. For example, ventral striatal activity might correlate with predicted utility in the ICR condition because that region is causally responsible for the sluggishness with which items can be removed from WM. By this account, ventral striatum might either enact reallocation, or conversely “veto” reallocation, whenever predicted utility of the putatively irrelevant information remains relatively high. There is some recent support for the latter possibility: direct electrical stimulation of the ventral striatum preserves the maintenance of action plans that are no longer necessary to receive a reward (Jurado-Parras et al., 2012), as might be expected if ventral striatum serves to delay or veto WM reallocation. However, future genetic, patient, and/or pharmacological interventions targeting dopaminergic action more directly will be required to confirm the role of dopamine in supporting the utility based reallocation and maintenance functions identified here.

Interestingly, the frontal correlates of predicted utility as observed in the RCI and RCR conditions were significant in a fairly caudal sector of dorsal PMd cortex (see Figure 5). No correlations with predicted utility were observed in the ICR condition within these dorsal PMd regions, even at extremely liberal thresholds (*p* < 0.05, uncorrected). Likewise, little correlation with predicted utility within the RCI condition was observed in the more immediately rostral frontal cortex (*p* < 0.05 uncorrected), though regions like pre-PMd and IFS were activated by the contrast of RCI/ICR vs. RCR. Hence, the apparent focality of the RCI/RCR correlation diverges from the much wider-spread results of our task-based contrast across conditions (see Figure 4). Such divergence might suggest that the utility of more concrete or lower-level items is particularly demanding on caudal frontal substrates. This possibility is intriguing in light of prior work suggesting systematic rostral vs. caudal differences in lateral frontal cortex as increasingly abstract rules and contexts are used to govern action (Koechlin et al., 2003; Badre and D'Esposito, 2007; Nee and Brown, 2012; Reynolds et al., 2012; Nee et al., 2013). This prior work has identified a PMd region, partially overlapping with the present focus, as particularly important for control according to simple rules that directly map a stimulus to a response. Thus, in the present task, it is striking that utility for the lower-level item appears to be tracked in this PMd region for conditions when the lower level items must be maintained as a direct context for response selection. Nevertheless, future work manipulating additional levels of abstraction would be required to assess whether the locus of effect here is due to the level of representation being maintained, and to provide the necessary region by effect interactions that would support such functional specificity.

Future work should also confront the potentially important relationship between utility and demands on selective attention. Notably, the simple difference in BOLD between the RCR and other conditions throughout frontoparietal cortex might reflect both selective attention (i.e., both ICR and RCI involve selectively attending to one particular class of lower-level items) as well as utility-based WM reallocation. However, the distinct correlations with utility observed between the ICR and other conditions suggest that, at a minimum, selective attention and utility interact; a conception of selective attention that would not be unlike the WM and gating functions we propose here. Future work should test whether selective attention can be engaged in a way that is truly independent of predicted utility, or whether they are causally related (e.g., predicted utility might set the selectivity of attention).

One alternative interpretation of our behavioral results is that the differential behavioral benefit of ISI reflects the compulsory closure of an input gate following the presentation of context in the ICR condition. We find this a dissatisfying explanation for the complete pattern of results for several reasons. First, gate closure would not be helpful in the ICR condition (where the next item is deterministically relevant). Second, it's unclear why gate closure would occur differentially in ICR condition relative to the RCI condition, where the next item is deterministically irrelevant and so gate closure would be helpful. Third, it's unclear why gate closure would be differentially related to predicted utility in the ICR condition relative to the other conditions, particularly the RCI condition. And, finally, it's unclear why the timecourse of this effect should be so much slower than other phenomena potentially arising from the compulsory closing of an input gate (e.g., the attentional blink; Olivers and Meeter, 2008).

A related factor that may interact with utility, and WM reallocation, is time. WM representations with temporally-delayed utility should undergo some discounting, so as to avoid opportunity costs: WM might otherwise remain occupied with items that are hugely useful but only for some distant opportunity. Discounting could help reallocate WM in these situations, so as to represent information of value in the nearer term. One simple mechanism would be to impose a minimum threshold on the utility of information that is to be maintained. Alternatively, more sophisticated discounting functions might be applied to the contents of WM. Future work should attempt to distinguish the functional form relating predicted utility to time, behavior and the consumption of WM resources.

As alluded to above, our work does not address the important—but distinct—question of what mechanism supports WM reallocation. Instead, we use the term “reallocation” more broadly, to refer a goal of the WM system that could be accomplished in many ways. For example, WM reallocation could occur through the indirect removal of non-utile items (e.g., via their decay or “sudden death”; Zhang and Luck, 2009) or through their indirect overwriting (e.g., via interference). Alternatively, WM reallocation could occur through more “direct” or targeted removal (e.g., via the deletion or clearing of a non-utile WM representation) or more targeted overwrite (e.g., via updating a specific non-utile representation with information that is more utile). WM reallocation could also occur through deprioritization, such as by shifting a capacity-limited “focus of attention” (Oberauer et al., 2012), or by closing an “output gate” to prohibit the influence of this maintained item on action (Chatham et al., under review). Of note, extant models of WM reallocation span this gamut of mutually-compatible possibilities, suggesting that all could be plausible mechanisms supporting this function (Hazy et al., 2006; O'Reilly and Frank, 2006; Oberauer and Lewandowsky, 2011; Oberauer et al., 2012).

Our study was not designed to distinguish among these alternative mechanisms. Nevertheless, there is at least one finding in this study that might be informative for future directed experiments and theory. Of note, we failed to locate a region that was differentially activated between the ICR and RCI conditions. This is perhaps surprising from the perspective of a targeted removal or deletion process, as one might have predicted that such a process would be associated with differential activation during the sole condition demanding removal (i.e., ICR). However, as this is a null result, we also cannot conclusively rule out the existence of targeted removal process from the present results.

A more direct implication of our model and data is that WM resources can be strategically reallocated in a way that correlates with basic RL principles. Trial-by-trial fluctuations in performance and frontoparietal and striatal BOLD can be predicted by a reinforcement learner tracking the utility of information in WM. These correlations suggest that RL might play a role not only in controlling the input to WM, but also in determining whether information should undergo continued maintenance. Such findings oblige theoretical models of WM to account for potential interactions with RL systems capable of tracking utility.

Relatedly, our results highlight a potential confound between WM load and predicted utility in standard WM paradigms. For example, predicted utility and WM load are often either intrinsically collinear (as in change detection and partial report procedures) or could be perceived as such (e.g., if a correct response requires N bits of maintained information, each bit might be associated with a utility of 1/N). Some effects thought to reflect WM load effects could actually arise from these differences in utility. Tasks where load and predicted utility are somewhat more orthogonal (e.g., n-back) might clarify this issue, but also involve numerous and oppositely-signed changes in utility of the information in WM at every trial. For this reason, future work may require models of the kind presented here in order to separate these demands.

### Conflict of interest statement

The authors declare that the research was conducted in the absence of any commercial or financial relationships that could be construed as a potential conflict of interest.

## Footnotes

^1^As noted previously, the elevated RT in the RCR condition may not exclusively reflect its increased WM demands. For example, the elevated RT in this condition could reflect extra demands on the deployment of external attention during the final stimulus display. Nonetheless, such demands on external attention are constant across the ISIs of each condition; they thus offer a less parsimonious explanation than WM load for the full pattern of effects.
